# Comparison of surgical and conservative treatment outcomes for type a aortic intramural hematoma

**DOI:** 10.1186/s13019-024-02533-0

**Published:** 2024-03-06

**Authors:** Li Yin, Jiankai Wang, Zhibing Qiu, Xin Chen, Cunhua Su

**Affiliations:** Department of Thoracic and Cardiovascular Surgery, Nanjing First Hospital, Nanjing Medical University, 68 Changle Road, Nanjing, 210006 Jiangsu People’s Republic of China

**Keywords:** Aortic intramural hematoma, Aortic dissection, Conservative treatment, Surgical treatment, Long-term mortality

## Abstract

**Objective:**

This study aimed to compare hospital and long-term clinical outcomes associated with various treatment methods for Stanford A type aortic intramural hematoma (IMH) to provide a reference for clinical decision-making.

**Methods:**

In this single-center cohort study, we retrospectively analyzed 73 patients with Type A IMH treated at our center from August 1, 2018 to August 1, 2021. Among these patients, 26 were treated conservatively, and 47 underwent surgical intervention. We next compared this IMH cohort with 154 patients with acute type A aortic dissection (AD) who were treated surgically during the same study period.

**Results:**

Computed tomography angiography revealed that the diameter of the ascending aorta of IMH patients treated with surgery was higher than IMH patients treated with conservative therapy (44.92 ± 7.58 mm vs. 51.22 ± 11.85 mm, *P* < 0.05), while there was no significant difference in other clinical parameters. The in-hospital mortality of patients with IMH who underwent surgical treatment was lower than those undergoing conservative treatment (0% vs. 11.5%, *P* < 0.05). The long-term mortality of the conservative IMH group was higher than the surgical IMH group (26.1% vs. 8.5%, *P* < 0.05). There was no significant difference in the surgical parameters and postoperative complications between AD and IMH surgery patients. The proportion of circulatory arrest time in the lower body (19.98 ± 9.39 min vs. 17.51 ± 3.97 min) and arch involvement (98 (63.6%) vs. 22 (46.8%)) in the IMH surgery group was lower than in the AD surgery group (*P* < 0.05).

**Conclusions:**

Compared with conservative treatment, surgical treatment of IMH significantly improves the survival rate of patients. Thus, surgical intervention should be considered the primary treatment option if feasible. Furthermore, The safety of IMH surgery can be guaranteed just like AD. But we still need in the future evidence on bigger samples.

**Graphical abstract:**

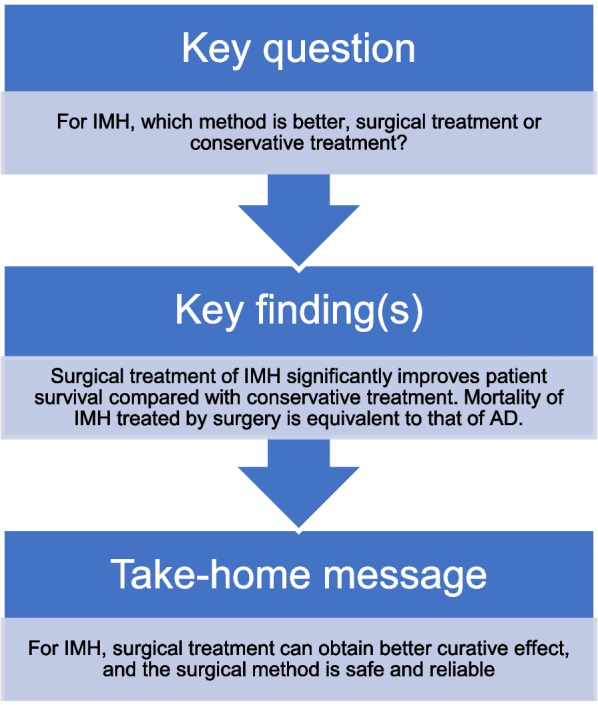

## Introduction

The pathological and physiological basis of AD is weakness in the middle layer of the aorta, cystic necrosis in the middle layer, rupture of arterial elastic fibers and smooth muscles, formation of fibrosis and hyaline degeneration. Due to lesions in the middle layer of the artery, the lumen expands and the adhesion between the intima and middle layer decreases. Under the action of internal and external forces, the intima tears and blood flows between the intima and middle layer, causing the intima and middle layer to peel off and develop in the circumferential and longitudinal directions, ultimately forming an aortic dissection. There is currently controversy about the mechanism of IMH, with mainstream theories including the rupture of nutrient vessels in arterial walls, penetrating ulcers, and small intimal tears [1].

Aortic Intramural hematoma (IMH) is a significant subtype of acute aortic syndrome (AAS), accounting for 10–20% of all AAS cases [2]. It is widely thought that IMH is a precursor to aortic dissection (AD), given that they share similar pathogenic factors, pathogenesis, and risk factors [3]. Like AD, IMH can be categorized into Stanford type A and Stanford type B according to the location of involvement. In China, there are well-defined guidelines to guide the treatment of various types of aortic dissection. Conservative treatment has been associated with poor outcomes for Stanford type A acute aortic dissection, making surgical intervention the primary treatment modality [3]. However, whether surgical treatment is necessary for type A IMH remains controversial within the medical community worldwide [4]. China has not yet established its guidelines for treating IMH, resulting in ongoing debates surrounding the optimal treatment approach, unlike AD. Advancements in medical imaging, such as computed tomography angiography (CTA), coupled with increased awareness of the disease, have led to a higher likelihood of early detection and diagnosis of aortic dissection cases, including patients with intramural hematoma. However, there is an ongoing debate about whether emergency surgical intervention should be conducted for patients with type A IMH due to the highly invasive nature of the procedure, which is associated with elevated postoperative mortality and complication rates.

The primary goals of open surgery for AD and IMH are to prevent (or treat) aortic rupture, prevent retrograde extension of the dissection to the aortic root, prevent antegrade spread of the dissection to distant but unresectable segments, and alleviate reperfusion syndrome. Surgical intervention can reduce the direct risk of aortic rupture/tamponade and reconstruct blood flow to inappropriate vessels [5].

The decision of whether to proceed with surgery for a patient diagnosed with intramural hematoma poses a challenging dilemma for surgeons. To assist in making informed decisions regarding emergency room patients with IMH, it is crucial to understand the mortality rates, complications, and overall patient benefits associated with conservative and surgical management approaches. This study compared the prognostic outcomes of conservative and surgical treatment and the perioperative outcomes of surgical treatment in IMH patients treated at the Cardiovascular Center of Nanjing First Hospital in the past 3 years toanalyze more suitable treatment methods for Chinese population.

## Methods

### Data collection

For this study, we conducted a retrospective review of the medical records of patients diagnosed with type A intramural hematoma who received either surgical or conservative treatment at the Cardiothoracic Surgery Department of the First Hospital of Nanjing, China. The data collection period spanned from August 1, 2018, to August 1, 2021. Specifically, we analyzed the inpatient and outpatient records of 26 IMH patients who received conservative treatment, 47 IMH patients who underwent surgical treatment, and 154 patients diagnosed with aortic dissection who underwent surgical treatment. The data used in this study were obtained from various sources, including clinical course records, examination reports, and the clinical database. Follow-up and supplementary data were also collected to ensure comprehensive information for the analysis. Given that this was an observational retrospective study, informed consent was not required. The inclusion criteria were as follows: 1. patients with Stanford type A IMH diagnosed by computed tomography angiography; 2. aged between 18 and 80 years old; 3. patients treated with conservative drug therapy or thoracotomy in our center. Exclusion criteria: 1. Patients with known underlying conditions, including Marfan syndrome, multiple nodular arteritis, systemic lupus erythematosus, etc.; 2. Patients with a history of drug abuse; 3. Patients with mental disorders, speech disorders, severe hearing impairment, and other factors that hinder normal communication; 4. Patients with underlying diseases such as malignant tumors and extremely poor health conditions.

### Conservative treatment and surgical technique

All patients diagnosed with type A IMH were placed on absolute bed rest and closely monitored for changes in vital signs. They received antihypertensive and negative inotropic drug treatment (such as beta-blockers combined with other antihypertensive drugs). Analgesia treatment was also provided for patients with severe symptoms to control blood pressure and heart rate. Our expected goal is to control systolic blood pressure below 140 mmHg and heart rate between 60 and 100 bpm. The criteria for the success of Conservative management is the reduction or disappearance of patients’ symptoms, improvement of pathological imaging findings, and a long-term good prognosis for patients. The decision to perform surgical treatment was based on a comprehensive assessment of the patient's aortic CTA, ultrasound findings, and symptoms.

Type A IMH often affects critical anatomical structures within the aortic root, such as the aortic valve, aortic sinus, and coronary artery orifice. The fundamental principle of surgical treatment is to achieve complete removal of the torn intima, ensuring proper blood supply to the coronary artery orifice and restoring normal functionality of the aortic valve. Surgeons perform total aortic arch replacement and frozen elephant trunk surgery to treat the part of the aorta. Additional procedures may be necessary based on the involvement of the aortic root and the specific extent of the condition, such as aortic valve replacement, Bentall operation, David operation, and other suitable interventions.

### Evaluation and follow up

Mortality in this study was defined as any death occurring either during the patient's hospital stay or after discharge including in-hospital mortality and long-term mortality, regardless of the cause. Complications were defined as specific adverse events that occurred within 30 days following surgery or conservative treatment. These complications included postoperative low cardiac output, the need for a second tracheal intubation, acute kidney injury, the requirement for a second thoracotomy, postoperative infections, brain complications, spinal cord complications, and poor healing of surgical incisions. All patients were followed up at multiple time points: 1 month, 3 months, 6 months, and 12 months after surgery or conservative treatment through outpatient examinations and telephone inquiries. Data on the survival status of the patients was collected to analyze the long-term mortality rate of patients.

### Statistical analysis

All data collation and statistical analyses were performed using the IBM SPSS statistical package (version 26.0, SPSS Inc, Chicago, USA). Continuous variables were tested for normality of distribution by mean ± standard deviation, and Student’s t-test was used for the normal distribution of variables. Count data were expressed as frequencies (percentages). The chi-square test was used for comparison between groups. The survival rates of the different treatment methods for type A IMH were analyzed using Kaplan–Meier and log-rank statistics. A *p*-value of less than 0.05 was considered statistically significant.

## Results

### Comparison of conservative and surgical treatment of IMH

**Fig. 1:**
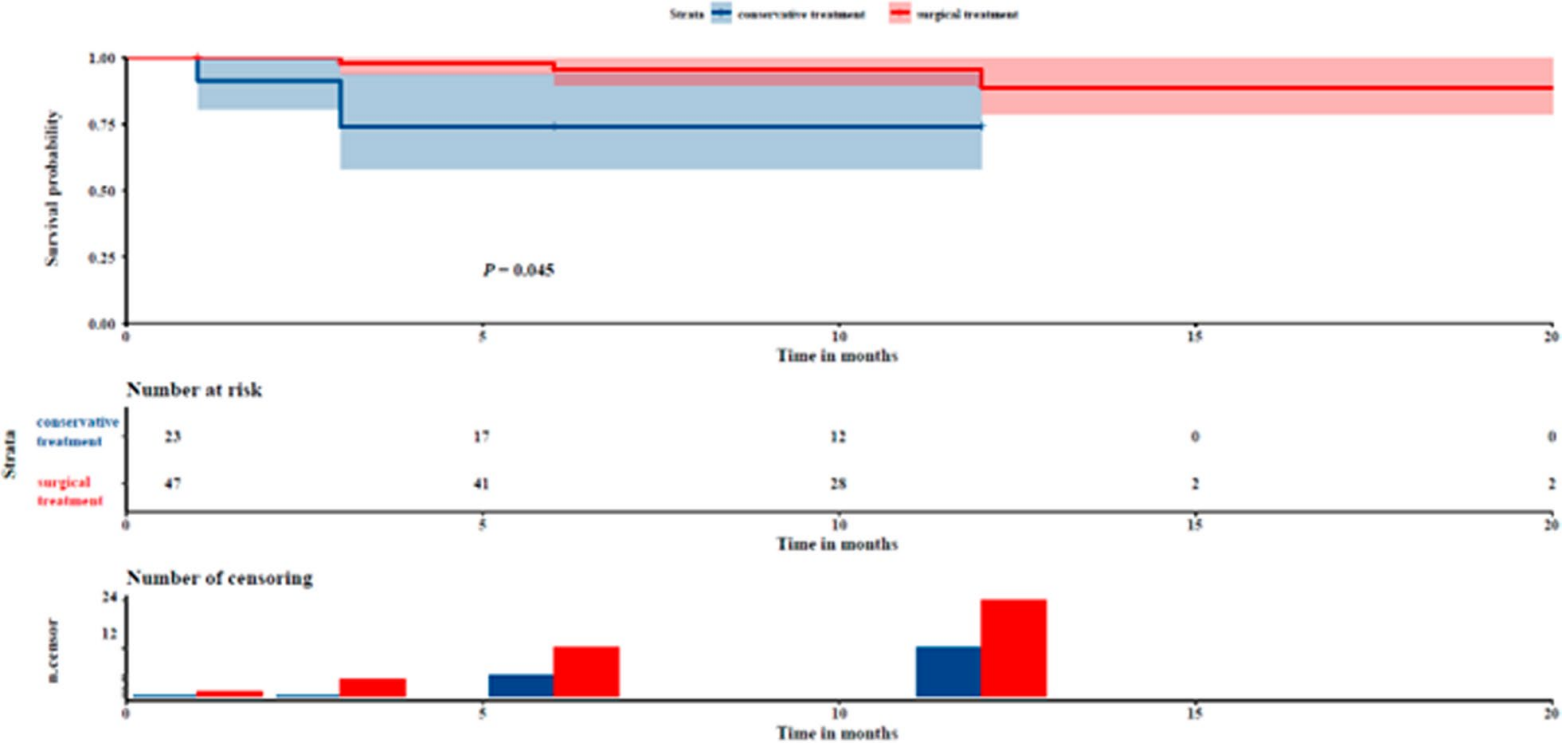
Long term mortality comparison between IMH undergoing surgical treatment and conservative treatment

The Student's t-test and chi-square test for two independent samples revealed no significant differences between the two groups in terms of gender, age, BMI, LVEF, smoking history,, etc. (*P* > 0.05). However, the admission CTA revealed that the ascending aortic diameter was higher in patients treated with IMH surgery than with conservative treatment, and the in-hospital mortality was lower in surgically treated IMH patients than those conservatively treated (*P* < 0.05)(Table 1). In the IMH conservative treatment group, there were 3 in-hospital deaths and 6 out-of-hospital deaths. On the other hand, there were no in-hospital deaths and 4 out-of-hospital deaths in the IMH surgical treatment group. The Kaplan–Meier analysis with the Log Rank (Mantel-Cox) test demonstrated that the mortality rate in the IMH conservative treatment group was significantly higher than in the IMH surgical treatment group (*P* < 0.05) (Fig. 1).

**Table 1:** Comparison of general data of patients with IMH undergoing surgical treatment and conservative treatment

Category	Conservative treatment (*n* = 26)	Surgical treatment (*n* = 47)	*χ*^2^/*t*	*P* value
Sex (male,%)	17 (65.4)	39 (83)	2.901	0.089
Age (yrs)	57.73 ± 9.48	54.36 ± 10.89	1.323	0.190
BMI (kg/m^2^)	25.65 ± 2.81	26.88 ± 3.62	− 1.601	0.114
LVEF (%)	57.19 ± 4.56	60.30 ± 7.21	− 1.984	0.051
Cerebrovascular accident (%)	2(7.7)	7(14.9)	0.275	0.600
Hemopericardium (%)	0(0)	8(17)	3.379	0.066
Smoking (%)	8(30.8)	21(44.7)	1.353	0.245
Diabetes (%)	6(23.1)	6(12.8)	0.654	0.419
Hypertension (%)	21(80.8)	42(89.4)	0.445	0.505
Hyperlipidemia (%)	2(7.7)	4(8.5)	–	1.000
COPD (%)	3(11.5)	3(6.4)	–	0.659
IMH maximum thickness (mm)	44.92 ± 7.58	51.22 ± 11.85	− 2.767	0.007
Diameter of ascending aorta hematoma (mm)	9.12 ± 4.19	10.90 ± 5.35	− 1.460	0.149
Hospital mortality (%)	3(11.5)	0(0)	–	0.042

*Cerebrovascular accident: Occlusion or hemorrhage of brain, spinal cord or retinal artery causes symptoms lasting for more than 24 h; COPD (Chronic obstructive pulmonary disease): Persistent airway obstruction caused by emphysema or chronic bronchitis; Diameter of ascending aorta and Diameter of ascending aorta hematoma: maximum aortic and aorta hematoma diameter of CTA

### Comparison of surgical treatment of AD and IMH

**Fig. 2:**
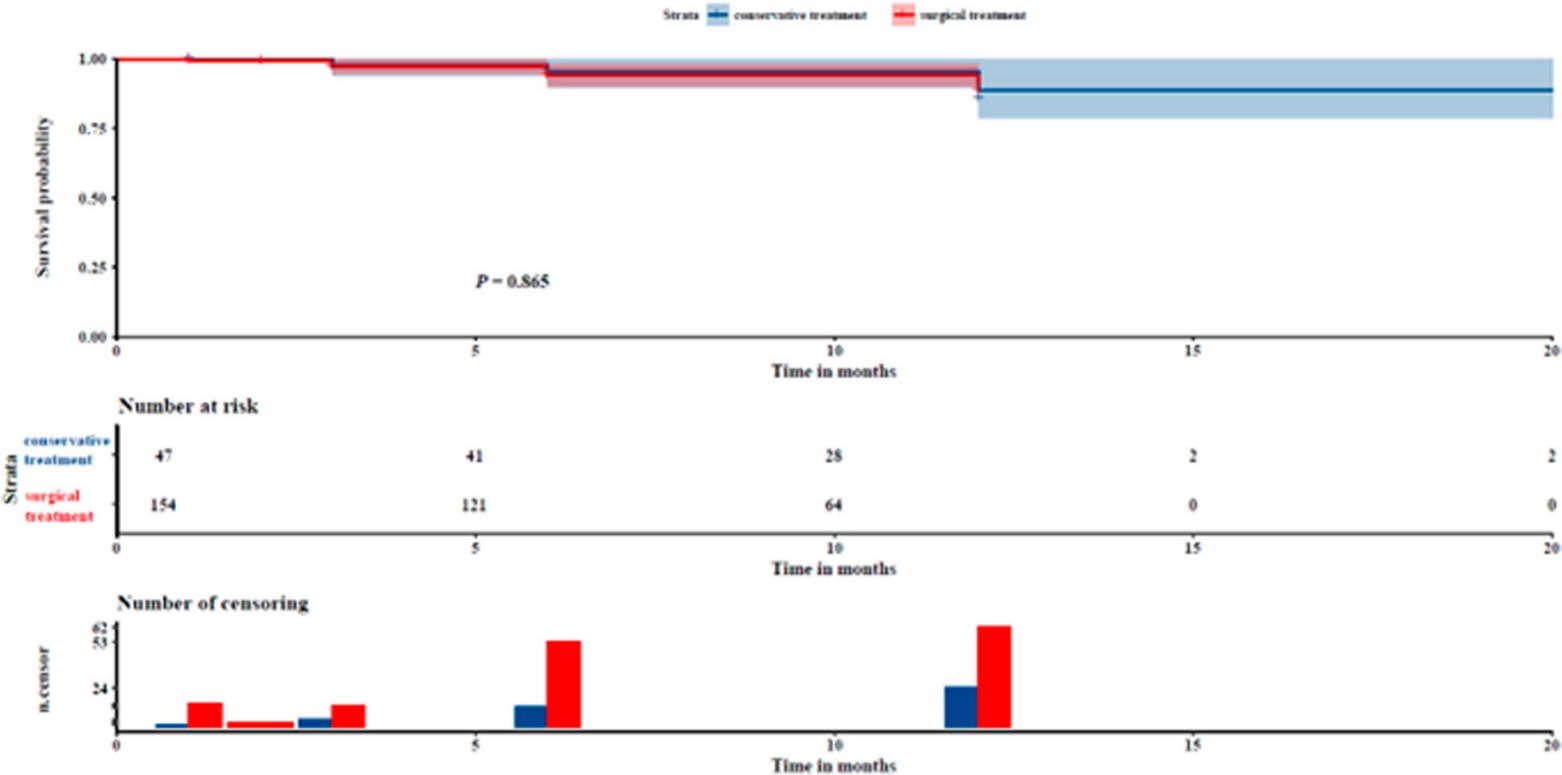
Long term mortality comparison between AD undergoing surgical treatment and IMH undergoing surgical treatment

The independent samples Student's t-test and Chi-square test were conducted to compare differences in gender, age, BMI, LVEF, Euro SCORE, time to aortic cross clamp, time to extracorporeal circulation, etc. There was no significant difference between groups in secondary tracheal intubation, acute kidney injury, secondary chest opening, infection, brain complications, spinal cord complications, and poor healing after median sternotomy within 30 days after surgery (*P* > 0.05). The percentage of patients in the IMH surgery group with lower body circulatory arrest and arch involvement (type C) was lower than that in the AD surgery group (*P* < 0.05) (Table 2). There were 10 out-hospital deaths in the AD surgery treatment group and 4 out-hospital deaths in the IMH surgery treatment group. The Log Rank (Mantel-Cox) test by the Kaplan–Meier method found no significant difference in mortality rates between the two groups (*P* > 0.05) (Fig. 2).

**Table 2:** Comparison of general data of IMH undergoing surgical treatment and AD undergoing surgical treatment

Category	Surgical treatment of AD (*n* = 154)	Surgical treatment of IMH (*n* = 47)	*χ*^2^/*t*	*P value*
Sex (male, %)	110 (71.4)	39 (83)	2.505	0.113
Age (yrs)	54.99 ± 12.33	54.36 ± 10.89	0.312	0.755
BMI (kg/m^2^)	26.09 ± 3.86	26.88 ± 3.62	1.250	0.213
Smoking (%)	64 (41.6)	21 (44.7)	0.144	0.704
Diabetes (%)	9 (5.8)	6 (12.8)	1.597	0.206
Hypertension (%)	127 (82.5)	42 (89.4)	1.279	0.258
Hyperlipidemia (%)	8 (5.2)	4 (8.5)	0.238	0.625
COPD (%)	7 (4.5)	3 (6.4)	0.015	0.901
LVEF (%)	61.29 ± 6.87	60.30 ± 7.21	0.853	0.395
Euro SCORE II	5.65 (3.55, 9.77)	5.66 (4.04, 11.02)	− 0.431	0.666
Aortic Cross-clamping time (min)	90 (76, 103)	89 (78, 106)	− 0.795	0.427
Circulatory arrest duration (min)	19.98 ± 9.39	17.51 ± 3.97	2.593	0.010
Cardiopulmonary bypass time (CPBT) (min)	165 (142.75, 186.25)	170 (152, 193)	− 1.274	0.203
Stay time of ICU(day)	3 (2, 6)	3 (2, 6)	− 0.161	0.872
Cerebrovascular accident (%)	30 (19.5)	7 (14.9)	0.504	0.478
Pericardial effusion (%)	25 (16.2)	8 (17)	0.016	0.898
Secondary block (%)	24 (15.6)	7 (14.9)	0.013	0.909
Cardioplegic solution (%)			2.106	0.349
Buckberg	14 (9.1)	4 (8.5)		
Del Nido	123 (79.9)	34 (72.3)		
HTK	17 (11)	9 (19.2)		
Further classification (%)			1.866	0.389
Type A1	110 (71.4)	31 (66)		
Type A2	29 (18.8)	8 (17)		
Type A3	15 (9.8)	8 (17)		
Aortic arch involvement (%)			4.238	0.040
Type C	98 (63.6)	22 (46.8)		
Type S	56 (36.4)	25 (53.2)		
Low cardiac output (%)	6 (3.9)	1 (2.1)	0.015	0.901
Secondary endotracheal intubation (%)	11 (7.1)	7 (14.9)	1.788	0.181
Acute kidney injury (%)	26 (16.9)	8 (17.0)	< 0.001	0.982
Second operation (%)	26 (16.9)	5 (10.6)	1.077	0.299
Infection (%)	48 (31.2)	9 (19.1)	2.561	0.110
Brain complications (%)	27 (17.5)	11 (23.4)	0.810	0.368
Spinal cord complications (%)	8 (5.2)	1 (2.1)	0.237	0.373
Poor wound healing (%)	10 (6.5)	5 (10.6)	0.396	0.529
Hospital mortality (%)	0 (0)	0 (0)	–	1.000

^*^Further classification: A1: normal sinus of valsalva, A2: mild lesion of sinus of valsalva, A3: severe lesion of sinus of Valsalva; Aortic arch involvement: Type C: The aortic arch is affected, Type S: The aortic arch is not affected; Low cardiac output: Cardiac index < 2.0 or Using IABP, Impella, ECMO and other ventricular assist devices; Brain complications: Including stroke, transient ischemic attack (TIA) and delirium; Spinal cord complications: Including paraplegia and sensory loss; Poor wound healing: All kinds of problems of chest incision need to be sutured again within 30 days after operation. Second operation means a second procedure including surgical hemostatic revision

### Univariate and multivariate COX regression analysis of distant mortality in surgical patients

A univariate COX regression analysis revealed that higher Euro SCORE, longer duration of aortic cross clamp and extracorporeal circulation, history of cerebrovascular accident, and presence of pericardial effusion were significant factors influencing the occurrence of death [HR = 1.074 (1.054, 1.095), *P* < 0.001; HR = 1.037 (1.023, 1.050), *P* < 0.001; HR = 1.012 (1.005, 1.018), *P* = 0.001; HR = 17.555 (4.892, 62.994), *P* < 0.001; HR = 13.922 (4.364, 44.406), *P* < 0.001]. Multifactorial COX regression analysis revealed that higher Euro SCORE, longer duration of aortic cross clamp, and presence of pericardial effusion were independent risk factors for the occurrence of death [HR = 1.049 (1.018, 1.080), *P* = 0.002; HR = 1.027 (1.006, 1.049), *P* = 0.010; HR = 6.000 (1.666, 21.617), *P* = 0.006] (Table 3).

**Table 3:** Univariate and multivariate COX regression analysis of long-term mortality in IMH and AD patients undergoing surgery treatment

Category	Univariate analysis			Multivariate multivariate		
	*β*	*HR* (95% CI)	*P value*	*β*	*HR* (95% CI)	*P value*
Sex	− 0.849	0.428 (0.149, 1.234)	0.116			
Age	0.022	1.022 (0.978, 1.068)	0.340			
Length	− 0.028	0.972 (0.919, 1.029)	0.331			
Weight	− 0.004	0.996 (0.961, 1.033)	0.837			
LVEF	− 0.013	0.987 (0.921, 1.058)	0.715			
Euro SCORE II	0.072	1.074 (1.054, 1.095)	< 0.001	0.048	1.049 (1.018, 1.080)	0.002
Aortic Cross-clamping time	0.036	1.037 (1.023, 1.050)	< 0.001	0.027	1.027 (1.006, 1.049)	0.010
Ischemia time of lower body	0.008	1.008 (0.957, 1.060)	0.771			
CPBT	0.012	1.012 (1.005, 1.018)	0.001	0.008	1.008 (0.999, 1.017)	0.079
Stay time of ICU	− 0.025	0.975 (0.873, 1.090)	0.657			
Cerebrovascular accident	2.865	17.555 (4.892, 62.994)	< 0.001	0.634	1.885 (0.325, 10.914)	0.479
pericardial effusion	2.633	13.922 (4.364, 44.406)	< 0.001	1.792	6.000 (1.666, 21.617)	0.006
Secondary block	0.432	1.540 (0.430, 5.524)	0.507			
Smoking	− 1.100	0.333 (0.093, 1.194)	0.091			
Diabetes	0.552	1.737 (0.389, 7.769)	0.470			
Hypertension	− 0.410	0.664 (0.185, 2.386)	0.530			
Hyperlipidemia	0.950	2.587 (0.579, 11.563)	0.214			
COPD	1.360	3.895 (0.863, 17.574)	0.077			
cardioplegic solution						
HTK		–	0.745			
Buckberg	− 12.461	< 0.001 (< 0.001, < 0.001)	0.984			
Del Nido	0.796	2.218 (0.290, 16.953)	0.443			
Further classification						
Type A3		–	0.569			
Type A1	− 0.351	0.704 (0.149, 3.316)	0.657			
Type A2	0.291	1.337 (0.245, 7.301)	0.737			
Aortic arch involvement	− 0.148	0.862 (0.299, 2.486)	0.784			
Low cardiac output	0.559	1.749 (0.228, 13.403)	0.591			
Secondary endotracheal intubation	0.932	2.539 (0.708, 9.108)	0.153			
AKI	− 0.148	0.863 (0.193, 3.855)	0.847			
Second operation	− 0.766	0.465 (0.061, 3.559)	0.461			
Infection	− 0.971	0.379 (0.085, 1.693)	0.204			
Neurological complications	− 0.381	0.683 (0.153, 3.052)	0.618			
Spinal cord complications	1.353	3.868 (0.865, 17.292)	0.077			
Poor wound healing	1.094	2.987 (0.832, 10.723)	0.093			

## Discussion

The exact pathogenesis of IMH is still not well understood. There are three main theories: firstly, the rupture of nutrient vessels within the middle layer of the aorta causes bleeding and hematoma formation, which expands outward; secondly, aortic wall weakness results in small ulcer formation, rupture and hematoma; and thirdly, some scholars consider IMH as a unique form of thrombotic dissection [6]. Furthermore, there is a viewpoint that type A IMH should be classified as thrombotic aortic dissection [7].

Different treatment guidelines for various types of aortic dissection have been established worldwide. However, China has yet to develop its own guidelines for treating IMH, leading to controversies regarding treatment plans. Internationally, different regions have developed distinct treatment guidelines for the management of IMH. The American College of Cardiology Foundation (ACCF), the Japanese Circulation Society (JCS), and the European Society of Cardiology (ESC) have issued different guidelines for the treatment of IMH [8–10]. In 2010, ACCF/JCS/ESC proposed guidelines for type A IMH, followed by additional guidelines in 2011 and 2014. In Europe and the United States, type A IMH is treated similarly to type A AD, emphasizing the need for early surgical intervention due to the associated risks. However, in Asian countries such as Japan and Korea, conservative treatment is indicated for uncomplicated type A IMH, and surgical treatment is indicated only for complicated type A IMH [11]. Sandhu Harleen K. revealed that a good preoperative evaluation and an active surgical plan helped improve the survival rate of IMH patients by comparing the different treatments of 101 IMH patients [12]. However, Tadashi Kitamura found no significant difference in early and midterm mortality between the conservative and surgical treatment of type A IMH. The diameter of the aorta, pain severity, and the presence of an aortic ulcer-like projection (ULP) were all factors that could influence the management of type A IMH. These factors play a significant role in determining the appropriate treatment approach for patients [13].

**Fig. 3:**
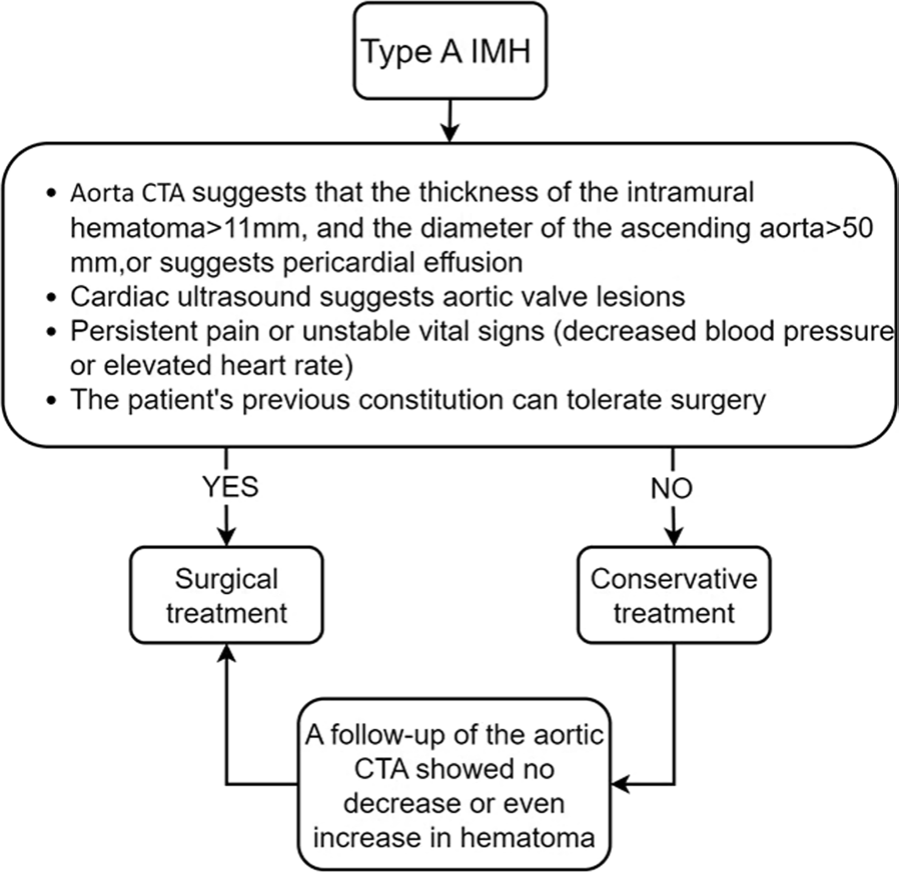
Diagnosis and treatment process of patients with acute type A IMH

There is currently no clear consensus on the treatment of type A IMH, which requires specific analysis based on the patient's condition and characteristics. This is also the purpose of our research on this topic. Considering the similarity in ethnicity, one might expect China to align with the treatment guidelines of Japan or other Asian countries. However, the current treatment approach for type A IMH in China leans towards active surgical intervention, similar to the practices of European and American countries. The selection of patients for surgery is primarily based on aortic CTA and cardiac ultrasound results, onset symptoms, and the overall physical condition of the patients. This approach is consistent with both domestic and international consensus. Our cardiovascular research center has developed its own treatment process based on relevant cases and studies from China and other countries in the world [14–16]. Active surgical intervention for type A IMH is considered if the aortic CTA suggests intermural hematoma thickness > 11 mm, maximum ascending aorta diameter > 50 mm, pericardial effusion or compression of coronary artery, or if the cardiac ultrasound suggests combined aortic valve lesion. In addition, if the patient's chest pain persists without relief or there are unstable vital signs (decreased blood pressure or increased heart rate), and the patient's previous physical condition can tolerate surgery, then active surgical intervention will also be considered. Conversely, conservative treatment will be chosen if these conditions are not met (Fig. 3).

Some studies have also shown that C-reactive protein (CRP) exhibits good performance in predicting the prognosis of IMH, while the increase of D-dimer level often indicates a poor prognosis of the disease. Due to the small sample size in the present study, further studies are required to validate these findings [17, 18]. However, if during the follow-up of conservatively treated patients, it is found that the patient's aortic intramural hematoma has not decreased or has even increased in size, or there are new risk factors, surgical treatment is indicated. It is now understood that the change in arterial diameter is the best predictor of whether acute IMH can be absorbed. A short-term increase in artery diameter is a high-risk factor for poor prognosis [19]. Some scholars advocate that ULP is a risk factor for IMH progress. In this respect, Ganaha et al. revealed that IMH caused by ULP tends to progress, while IMH without ULP is usually relatively stable [20].

**Fig. 4:**
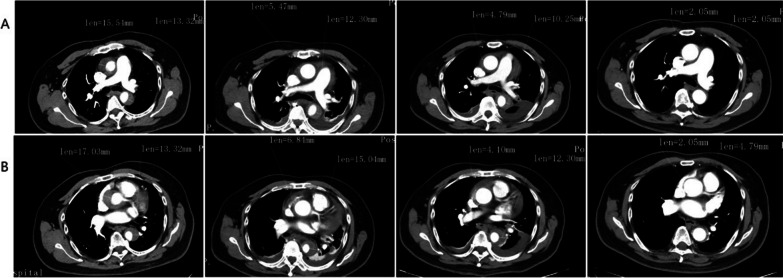
A typical case: CTA dynamic follow-up image of type A IMH patients undergoing conservative treatment. **a** The CTA images of pulmonary artery bifurcation plane at 1 month, 3 month, 6 month, and 12 month of the disease. **b** The CTA images of the left coronary artery orifice plane are 1 month, 3 month, 6 month, and 12 month

Patients that undergo conservative treatment are usually required to have a repeat aortic CTA 3 days, 1 week, 1 month, and 3 months after discharge. If the hematoma gradually decreases and the patient's symptoms are relieved, conservative treatment is considered effective and is continued, including effective analgesia, controlling heart rate and blood pressure, reducing aortic shear stress, and reducing the risk of aortic rupture. Importantly, using opioids (morphine) for analgesia can reduce the rise of blood pressure and heart rate caused by sympathetic nerve excitation. β-blocker (metoprolol) is the first-line drug for blood pressure control, which can ensure the lowest effective end-organ perfusion. If blood pressure is difficult to control, it must be combined with other drugs. The goal of drug treatment is to control the systolic blood pressure at 100–120 mmHg (1 mmHg = 0.133 kPa) and the heart rate at 60–80 times/min. In a typical case, following standard conservative treatment, significant reduction in the size of the aortic intramural hematoma and the largest diameter of the ascending aorta can be observed on CTA, indicating a tendency towards stability (Fig. 4). However, if upon review the hematoma does not decrease or even increases in size, surgical treatment will be recommended. It is important to note that the decision to pursue conservative treatment carries a certain risk of progression, as there have been reports in the literature of rapid progression from type A IMH to AD. Therefore, careful evaluation and monitoring of the patient’s condition is essential in determining the appropriate treatment approach. Good follow-up compliance and the surgeon's decisive judgment play a decisive role in the prognosis of patients, which requires the patient’s cooperation and the surgeon's experience from a large number of clinical cases. The surgical approach is selected according to the extent of IMH hematoma involvement and the location of the visible breach on intraoperative exploration, similar to AD. Patients with type A IMH exhibit long-term mortality rates that are not statistically different from those with classic AD, indicating that surgical treatment is a safe and reliable approach. These findings align with the research published by Kevin M in Circulation in 2012. However, Kevin M et al. noted that IMH is often accompanied by complications such as pericardial effusions and periaortic hematoma [21].

In the present study, univariate COX regression analysis revealed that higher EuroSCORE II, longer aortic cross clamping timeaortic cross clamp and extracorporeal circulation time, history of cerebrovascular accident, and history of pericardial effusion were important factors influencing the occurrence of death. The EuroSCORE II score is a relatively simple and highly accurate preoperative cardiac risk factor scoring method that can evaluate patients preoperatively based on patient- and cardiac-related factors. An accurate preoperative EuroSCORE II score helps surgeons to assess surgical risk more objectively and helps to guide the choice of the treatment plan. As the duration of aortic cross clamp and extracorporeal circulation increased, there was a greater need for effective myocardial protection, and the risk of severe cellular damage also increased. Therefore, choosing a rational surgical approach to minimize the duration of aortic cross clamp and extracorporeal circulation and selecting appropriate cardiac perfusion-stopping fluid can help improve the patient’s postoperative recovery. Most procedures for IMH involve treatment of the aortic arch, which necessitates unilateral cerebral perfusion during certain parts of the operation. This poses a challenge for ensuring cerebral protection. In our center, we routinely utilize ice caps to lower the temperature and reduce oxygen consumption intraoperatively. Additionally, we employed electroencephalogram (EEG) sensors to dynamically monitor EEG activity and ensure optimal cerebral protection. Patients with a preoperative history of combined cerebrovascular accidents have a higher likelihood of postoperative cerebral complications, which can affect overall mortality. Some IMH patients develop pericardial effusion preoperatively due to extravasation of blood from the hematoma into the pericardial cavity. A large amount of pericardial effusion can cause pericardial tamponade, which limits the systolic and diastolic function of the heart. Pericardial effusion caused by tearing of the endothelium near the coronary opening can affect the blood supply to the coronary arteries and cause acute myocardial infarction in severe cases. Accordingly, patients with preoperative combined pericardial effusion are at increased risk for surgery, conducive to a poor prognosis.

## Conclusion

Our study substantiated that surgical treatment of IMH significantly improves patient survival compared with conservative treatment and that surgical intervention is the first choice when the patient's physical condition and surgical conditions allow. Compared to the outcomes of surgical treatment for AD, surgical treatment of IMH provides a comparable level of safety. Preoperative Euro SCORE II, duration of aortic cross clamp and extracorporeal circulation, history of cerebrovascular accident, and pericardial effusion are independent predictors for postoperative mortality.

## Limitation

This study has several limitations, including the relatively small number of cases analyzed and the retrospective nature of the analysis, which may introduce inherent limitations and biases. Therefore, to establish the optimal treatment strategy for type A IMH, further validation is required through prospective studies with larger sample sizes involving multiple centers.

## Data Availability

The original contributions presented in the study are included in the article; further inquiries can be directed to the corresponding authors.

## Abbreviations

IMH: Intramural hematoma
AD: Aortic dissection
AAS: Acute aortic syndrome
CTA: Computed tomography angiography
BMI: Body mass index
LVEF: Left ventricular ejection fraction
ACCF: American College of Cardiology Foundation
JCS: Japanese Circulation Society
ESC: European Society of Cardiology
ULP: Ulcer-like projection
CRP: C-reactive protein
EEG: Electroencephalogram
